# Evaluating the Whole Patient: Lessons from the Pre-CKM Era Toward Integrated Cardio–Kidney–Liver–Metabolic Care

**DOI:** 10.3390/life16030492

**Published:** 2026-03-17

**Authors:** Felicia Chantal Derendinger, Annina Salome Vischer, Michael Mayr, Lilian Sewing, Isabelle Arnet, Thilo Burkard

**Affiliations:** 1 Medical Outpatient Department and Hypertension Center, ESH Hypertension Center of Excellence, University Hospital Basel, 4031 Basel, Switzerlandannina.vischer@usb.ch (A.S.V.); michael.mayr@usb.ch (M.M.); lilian.sewing@usb.ch (L.S.); 2 Medical Faculty, University of Basel, 4031 Basel, Switzerland; 3 Pharmaceutical Care Research Group, Department of Pharmaceutical Sciences, University of Basel, 4031 Basel, Switzerland; isabelle.arnet@unibas.ch; 4 Department of Cardiology, University Hospital Basel, 4031 Basel, Switzerland

**Keywords:** cardio–kidney–metabolic syndrome, CKM-syndrome, cardio–kidney–liver–metabolic syndrome, arterial hypertension, dyslipidemia

## Abstract

Before the American Heart Association introduced the cardiovascular–kidney–metabolic (CKM) syndrome concept in 2023, clinical care was largely organ-specific. This retrospective study analyzed diagnostic patterns and gaps in 406 patients with hypertension referred to and evaluated at the University Hospital Basel Hypertension Centre in 2017, 2019, or 2022 to identify blind spots in the assessment of cardio–kidney–liver–metabolic health. Electronic health records were used to assess CKM-relevant diagnostics, including lipid profiles, N-terminal pro-B-type natriuretic peptide (NT-proBNP), echocardiography, kidney function (estimated glomerular filtration rate: eGFR, urinary albumin-to-creatinine ratio: uACR), and hepatic assessment (Fib-4 score, abdominal ultrasound). Previously undetected conditions were identified according to contemporary criteria for dyslipidemia, chronic kidney disease (CKD), suspected heart failure (HF), diabetes, and suspected metabolic dysfunction-associated steatotic liver disease (MASLD). Although 94% of participants had laboratory data, key CKM parameters were inconsistently assessed. Of the participants, 39% had neither NT-proBNP measurement nor echocardiography, and 27% lacked hepatic ultrasound or sufficient data for Fib-4 calculation. Previously unrecognized comorbidities were common (suspected HF 21%, CKD 6%, suspected MASLD 3%). Lipoprotein(a) testing increased from 0% in 2017 to 23.7% in 2022, indicating growing awareness. Despite specialized care, diagnostic fragmentation persisted, underlining the need for systematic, interdisciplinary screening and informing the design of prospective registries such as the Swiss CKLM Registry to integrate patient-centered cardio–kidney–liver–metabolic care.

## 1. Introduction

Hypertension is the leading modifiable risk factor contributing to global all-cause morbidity and mortality, especially from cardiovascular disease (CVD) [1]. Its pathophysiology is closely linked to metabolic and kidney dysfunction, with obesity, insulin resistance, and dyslipidemia contributing to blood pressure elevation, cardiovascular risk and target-organ damage [2,3,4]. As a consequence, many patients with hypertension exhibit early or established abnormalities across cardiovascular, renal, metabolic, and hepatic systems, even when only one condition is clinically apparent [4,5,6,7]. Recognizing this interconnectedness, the American Heart Association (AHA) recently introduced the concept of cardiovascular–kidney–metabolic (CKM) syndrome, proposing a unified clinical framework that reflects the continuum of multi-organ dysfunction rather than isolated disease entities [5,6,7]. In the United States, more than one in four adults already fulfill criteria for at least one component of the CKM triad, underscoring the magnitude of the problem [6]. This integrative perspective has been further expanded by growing evidence that liver dysfunction, especially metabolic dysfunction-associated steatotic liver disease (MASLD), plays an important role in amplifying metabolic and cardiovascular risk [8,9,10,11,12].

Parallel to this conceptual shift, new therapeutic options with cross-organ benefits have transformed clinical management. Sodium–glucose cotransporter-2 (SGLT2) inhibitors, glucagon-like peptide-1 (GLP-1) receptor agonists and finerenone show clinically meaningful cardioprotective and renoprotective effects independent of their original indications [7,13]. Because eligibility for these agents depends on the timely identification of organ dysfunction, such as early heart failure, CKD, MASLD, or atherogenic dyslipidemia, systematic screening has become not only diagnostically relevant, but therapeutically necessary. Failure to detect subclinical disorders increasingly represents a missed opportunity for organ protection.

Although awareness of CKM syndrome and its holistic implications has increased, clinical practice has not kept pace, and many patients continue to be assessed within narrowly defined organ-based silos. Screening practices for, e.g., Lipoprotein(a), N-terminal pro–B-type natriuretic peptide (NT-proBNP), albuminuria, or liver fibrosis vary widely, and comprehensive evaluation across all CKM-related organ systems remains inconsistent in routine care [7,14,15,16,17]. Recent reports suggest that even in specialized tertiary settings, substantial proportions of patients may have undiagnosed organ dysfunction, such as heart failure, unless systematic screening is performed [18,19].

However, little is known about how comprehensively patients were evaluated before the CKM concept emerged, particularly in Europe, and how much diagnostic heterogeneity existed during the pre-CKM era. Understanding these early diagnostic patterns is crucial, as they uncover blind spots in routine care and at the same time establish the baseline needed to build coherent, integrated CKM screening strategies.

This study aimed to quantify the extent of CKM-related screening and diagnostic evaluation in hypertensive patients seen at a tertiary hypertension center in Switzerland in 2017, 2019, and 2022, before the introduction of the CKM framework and multi-organ protective therapies. We assessed whether key cardiovascular, kidney, metabolic, and hepatic parameters were evaluated, quantified non-assessed organ systems, and identified previously unrecognized CKM-related conditions. These insights informed the development of the prospective Swiss CKLM registry to support structured, multi-organ, patient-centered care.

## 2. Materials and Methods

### 2.1. Ethics

This study protocol was approved by the local ethics committee, Ethikkommission Nordwest- und Zentralschweiz (Ethics Commission Northwest and Central Switzerland, EKNZ 2025-00022). Participants were enrolled when general informed consent to use anonymized clinical and laboratory data for research purposes of the University Hospital Basel was either signed or not rejected by the participants.

### 2.2. Study Procedures, Design and Definitions

This retrospective analysis was based on data from a pre-existing database of 412 participants enrolled in the Single-Pill Combination (SPC) study (EKNZ 2023-00169). Participants were included if they had provided signed general consent or had not explicitly rejected it, and if they attended their first consultation at the tertiary Hypertension Centre of the University Hospital Basel in 2017, 2019, or 2022. These time periods were defined to assess progress in the use of antihypertensive single-pill combinations before and after the general recommendations within the hypertension guidelines by the European Society of Hypertension and European Society of Cardiology in 2018 [20]. All participants had a documented diagnosis of hypertension (defined as attended office blood pressure measurements of systolic ≥ 140 and/or diastolic ≥ 90 mmHg) [20] or were prescribed at least one antihypertensive medication, as recorded in their medical history. Additionally, participants had to be at least 18 years of age at the time of their first consultation. Exclusion criteria included individuals who rejected the general consent, were pregnant at the time of consultation, or had a consultation due to postpartum hypertension. Six participants from the database were excluded due to either withdrawal of the general consent of the University Hospital Basel in the meantime or duplicate registration. This resulted in a final study population of 406 individuals. Retrospective data supplementation was conducted for these participants with additional clinical and laboratory data from the local patients’ electronic health records. This included clinical history, documented risk factors for CKM syndrome, laboratory findings, and diagnostic data from transthoracic echocardiography (TTE) or abdominal sonography.

Initial consultation (IC) data were considered valid if collected within one month before or after the initial consultation. Data collection time points were defined at approximately 6 and 12 months, with data accepted within one month before or after the 6-month time point and within two months before or after the 12-month time point.

For diagnostic technical examinations, such as transthoracic echocardiography (TTE) and abdominal sonography, no strict time windows were applied. At initial consultation, examinations performed within one month before or after the index visit were included. At the 6- and 12-month data collection time points (TPs), technical examinations were accepted if performed within the respective one-year observation period, reflecting real-world scheduling outside predefined cut-offs. Accordingly, technical examination data were analyzed both at initial consultation and during data collection time points without strict temporal restrictions.

Diagnostic assessments were considered “performed” when the respective test was documented during the index evaluation at the hypertension center. “Not assessed” indicates that the specific parameter was not measured during the index evaluation at our center. In some cases, testing may not have been repeated because the respective condition had been previously diagnosed and documented in the medical record. Pathological findings were defined according to guideline-based thresholds for each parameter. Therefore, “not assessed” does not necessarily indicate absence of prior evaluation but reflects testing not performed or not repeated during the index assessment.

### 2.3. Definitions of Initial Consultation Characteristics

A positive family history of cardiovascular disease (CVD) was defined as CVD in a first-degree male relative before the age of 55 years or in a first-degree female relative before the age of 65 years. Medical treatment was characterized by medication burden, with total medication burden defined as the number of prescribed medication classes, antihypertensive medication burden as the number of antihypertensive drug classes, and the number of antihypertensive pills as the actual daily pill count of antihypertensive drugs.

### 2.4. Automated Office Blood Pressure

Blood pressure was routinely assessed during hypertension clinic visits using unattended automated office blood pressure (AOBP). To conduct AOBP, patients were instructed to sit quietly and alone in a room, maintaining an upright posture with feet flat on the floor and legs uncrossed. The blood pressure cuff, selected according to the individual’s arm size, was placed at heart level. Thereafter, clinical staff initiated the measurement sequence with an automated blood pressure measuring device and left the room. The device was pre-programmed to take three readings at 5, 7, and 9 min, which were then averaged automatically by the device. For AOBP blood pressure was classified as non-hypertensive if the average systolic value was <135 mmHg and the diastolic < 85 mmHg [20].

### 2.5. Definitions of Components of CKM Syndrome and Previously Undetected Conditions

The following definitions were applied to identify previously clinically undetected disorders in the study population:

### 2.6. Hypercholesterolemia

Undetected hypercholesterolemia was defined as a low-density lipoprotein cholesterol (LDL-c) level > 3.0 mmol/L [21] in individuals without a known history of dyslipidemia and not currently receiving lipid-lowering therapy such as statins, ezetimibe, proprotein convertase subtilisin/kexin type 9 (PCSK9) inhibitors, fibrates, or bempedoic acid.

### 2.7. Elevated Lipoprotein(a)

Elevated lipoprotein(a) levels were defined using the following cut-offs: values below 75 nmol/L were considered to contribute not additionally to increased cardiovascular risk, while values above 125 nmol/L were used to identify participants at increased cardiovascular risk. Measurements between 75 and 125 nmol/L were considered a gray zone [14,22]. LDL-c and lipoprotein(a) values were analyzed separately, as guideline recommendations to screen for lipoprotein(a) started in 2019.

### 2.8. Previously Undetected Suspected Heart Failure

Previously undetected suspected heart failure was defined based on one of the following criteria:

- H2fPEF-Score ≥ 5;
- Left ventricular ejection fraction (LVEF) < 53% for women and <51% for men;
- Left ventricular mass index (LVMI) > 95 g/m^2^ for women and >115 g/m^2^ for men;
- E-wave/A-wave ratio > 0.8, with at least two of the following conditions present:
  ◦ E/e’ ratio > 14 or;
  ◦ Tricuspid regurgitation (TR) velocity > 2.8 m/s;
  ◦ Left atrial volume index (LAVI) > 34 mL/m^2^;
  ◦ NT-proBNP > 125 pg/mL [23].

Participants meeting these criteria were considered to have previously undetected suspected heart failure, with no prior diagnosis or documented history of heart failure. NT-proBNP measurements between >75 and ≤125 pg/mL indicated possible cardiac stress, while values >125 pg/mL suggested previously undetected suspected heart failure [24].

### 2.9. Diabetes Mellitus

Undetected diabetes was defined as having an HbA1c level ≥ 6.5% or a random glucose measurement ≥ 11.1 mmol/L, in the absence of a documented history of diabetes or the use of anti-diabetic drugs, including metformin, insulin, dipeptidyl peptidase 4 (DPP-4) inhibitors, or sulfonylureas.

Prediabetes was defined as having an HbA1c level ≥ 5.7% or a glucose measurement ≥ 7.8 mmol/L, in the absence of a known history of diabetes or prediabetes, diabetes medication or undetected diabetes mellitus.

### 2.10. Previously Undetected Suspected Metabolic Dysfunction-Associated Steatotic Liver Disease (MASLD)

Previously undetected suspected MASLD was defined by the presence of hepatic steatosis on abdominal sonography in individuals without a known history of MASLD. Alternatively, a Fibrosis-4 (Fib-4) score > 3.25 [25], which can indicate advanced liver fibrosis and help rule in significant fibrosis, was also considered consistent with previously undetected suspected MASLD. The Fib-4 score was calculated using the patient’s age, levels of aspartate aminotransferase (AST) and alanine aminotransferase (ALT), and platelet count [26].

### 2.11. Chronic Kidney Disease (CKD)

Undetected CKD was defined based on the KDIGO (Kidney Disease: Improving Global Outcomes) guidelines for moderately elevated cardiovascular risk, provided there was no known history of CKD. Specifically, CKD was considered present in individuals with microalbuminuria, defined as an albumin-to-creatinine ratio (ACR) ≥ 3 mg/mmol. In the absence of microalbuminuria, undetected CKD was diagnosed if the estimated glomerular filtration rate (eGFR) was <60 mL/min/1.73 m^2^. This approach allowed for the identification of individuals with undiagnosed early-stage kidney dysfunction [27].

### 2.12. Statistical Methods

Continuous data were reported as mean ± standard deviation (SD) and categorical variables are described as counts (percents). The entire cohort was analyzed as described above. All statistical calculations were performed using R version 4.1.3.

## 3. Results

### 3.1. Initial Consultation Characteristics

We enrolled a total of 406 participants in the study. Of these, 119 (29.3%) participants were first seen in 2017, 131 (32.3%) in 2019, and 156 (38.4%) in 2022. Initial consultation characteristics are summarized in Table 1. Mean age was 57 years (standard deviation (SD) 15.6) and 56.4% of the study population were men.

### 3.2. CKM-Related Parameters Not Assessed in Routine Care

#### 3.2.1. Cardiovascular Parameters

**Figure 1:**
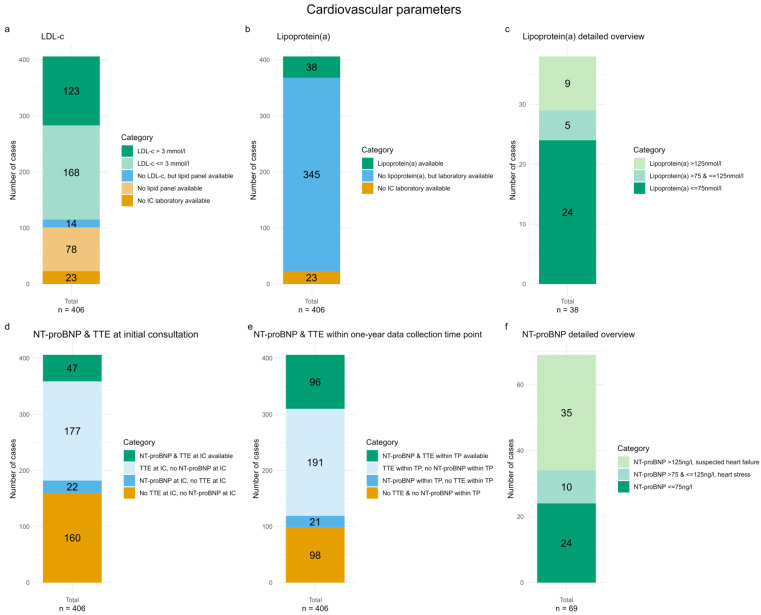
Bar charts showing the proportion of participants with an initial consultation (IC) laboratory test, lipid panel and LDL-c measurements, and the distribution of LDL-c categories (**a**); with and without lipoprotein(a) testing at IC (**b**); lipoprotein(a) levels among the 38 participants with IC measurement (**c**); with and without NT-proBNP measurement and transthoracic echocardiography (TTE) at IC (**d**) and within the one-year data collection time point (TP) (**e**); and the 69 participants who had an NT-proBNP measurement at IC (**f**). Detailed definitions are provided in Section 2.

In our analysis, 383 participants (94.3%) had laboratory data within the electronic health record available at IC; 23 participants (5.7%) had no laboratory testing at all. In total, 78 participants (19.2%) underwent laboratory testing without a lipid panel. Overall, LDL-c was available in 291 participants (71.7%), of which 123 (42.3%) were found to have a LDL-c level > 3mmol/L (Figure 1a). Lipoprotein(a) was available in 38 participants (9.4%), of which 9 (23.7%) were found to have an elevated cardiovascular risk, defined as a value > 125 nmol/L (Figure 1b,c). Lipoprotein(a) was tested in 0 out of 119 participants in 2017, 1 out of 131 participants (0.8%) in 2019, and 37 out of 156 participants (23.7%) in 2022.

Heart failure parameters, including TTE and NT-proBNP, were both assessed at IC in 47 participants (11.6%). In contrast, 160 participants (39.4%) had neither of these assessments available at IC (Figure 1d). We further evaluated whether some participants had an available NT-proBNP measurement or a TTE during the one-year data collection time point (TP) period. In total, 98 participants (24.1%) had neither an available NT-proBNP measurement, nor a TTE during the TP period. An additional 62 participants (15.3%) received at least one of these two examinations within the one-year TP (Figure 1e). A total of 69 participants (17.0%) had an available NT-proBNP measurement at IC (Figure 1f). More information about the assessment and pathological findings of diagnostic parameters can be found in Appendix A (Table A1).

#### 3.2.2. Kidney Parameters

**Figure 2:**
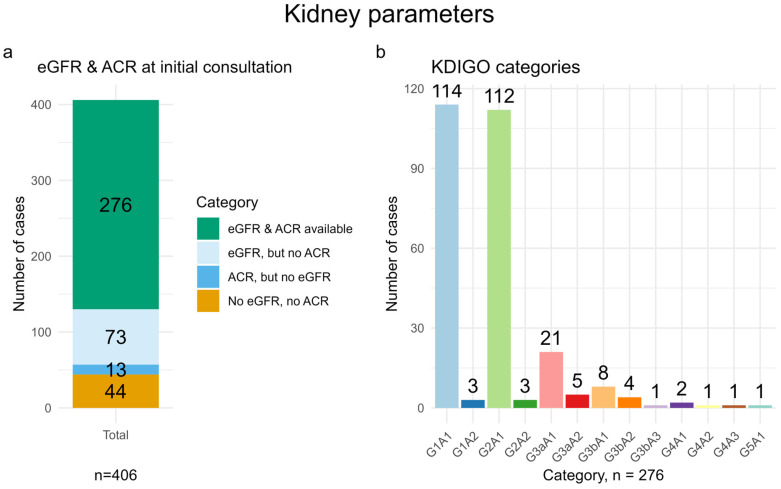
Bar charts showing the proportion of participants with and without eGFR and ACR measurements at initial consultation (IC) (**a**), and the KDIGO categories of the 276 participants who had both eGFR and ACR measurements at IC (**b**). Detailed definitions are provided in Section 2.

Both kidney parameters, eGFR and ACR, were missing in 44 participants (10.8%), while both parameters were available in 276 participants (68.0%) (Figure 2a).

Regarding KDIGO categories, 114 participants (28.1%) fell into category G1A1 and 112 participants (27.6%) into category G2A1. In total, 226 of the 276 participants with an available eGFR and ACR (81.9%) fell into the low-risk category and 27 participants (9.8%) into the moderate-risk category according to the KDIGO classification (Figure 2b).

#### 3.2.3. Metabolic Parameters Including Liver Parameters

**Figure 3:**
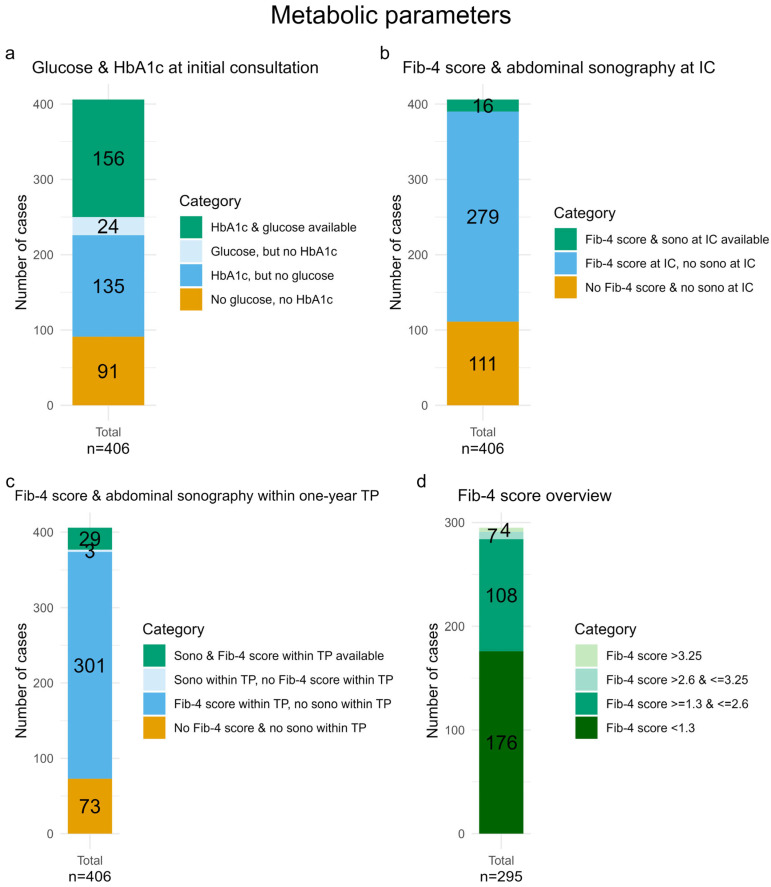
Bar charts showing participants with and without glucose and HbA1c measurements at initial consultation (IC) (**a**); with and without Fib-4 score and abdominal sonography at IC (**b**) and within a one-year data collection time point (TP) (**c**); and the distribution of Fib-4 scores at IC (**d**). Detailed definitions are provided in Section 2.

For the parameters related to diabetes mellitus, 91 participants (22.4%) had neither HbA1c nor blood glucose accessible, while both parameters were available in 156 participants (38.4%) (Figure 3a).

A Fib-4 score was possible to calculate in 295 participants (72.7%), whereas 119 participants (29.3%) had a calculated Fib-4 score > 1.3. Four participants (1.0%) had a Fib-4 score > 3.25 (Figure 3d).

Regarding the assessment of MASLD, 111 participants (27.3%) had neither abdominal sonography, nor all parameters to calculate the Fib-4 score accessible. At IC, 279 participants (68.7%) had a calculated Fib-4 score but no abdominal sonography. Consequently, only 16 participants (3.9%) had abdominal sonography at IC (Figure 3b). Four participants (3.4%), out of 119 participants with a Fib-4 score of 1.3 or above, had an abdominal sonography.

During the one-year TP, 73 participants (18.0%) had neither an abdominal sonography nor sufficient data to calculate the Fib-4 score, 38 fewer than at IC. In contrast, 32 participants (7.9%) received an abdominal ultrasound during TP, representing an increase of 16 participants (3.9%) compared to IC (Figure 3c).

### 3.3. Previously Undetected Conditions

#### 3.3.1. Cardiovascular

**Figure 4:**
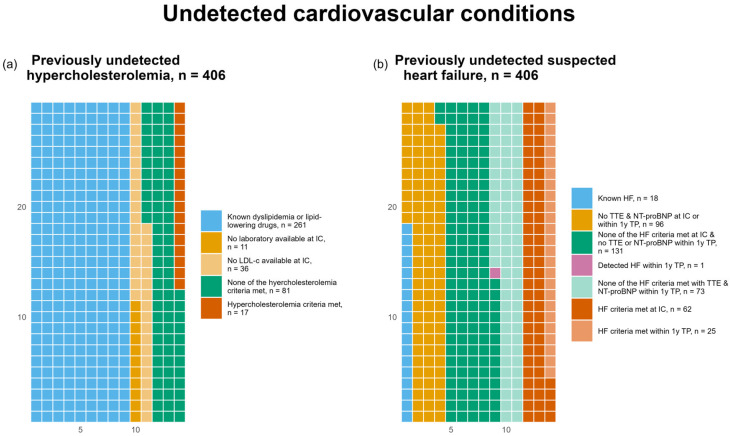
Waffle charts illustrating previously undetected hypercholesterolemia according to the predefined criteria (**a**), and previously undetected suspected heart failure (HF), stratified by detection at initial consultation (IC) or at the one-year data collection time point (1y TP) (**b**). Detailed definitions are provided in Section 2.

We identified 17 cases (4.2%) of previously undetected hypercholesterolemia. In contrast, 261 participants (64.3%) were already known to have dyslipidemia or were taking lipid-lowering medication. In total, 36 participants (8.9%) had an available laboratory at IC but no available LDL-c measurement, and most of them had elevated triglycerides levels, which limited the calculation of LDL-c (Figure 4a).

We found 87 participants (21.4%) with previously undetected suspected heart failure within the one-year TP, consisting of 62 cases (15.3%) at IC and 25 cases (6.2%) during the one-year TP. At IC, 18 participants (4.4%) had a previous diagnosis of heart failure. Meanwhile during the one-year TP one participant (0.2%) was diagnosed with clinically apparent heart failure during the regular clinical visits, who had no known HF at IC and no previously undetected HF at IC due to the HF criteria (Figure 4b). More information about previously undetected conditions can be found in Appendix A (Table A2).

#### 3.3.2. Kidney

**Figure 5:**
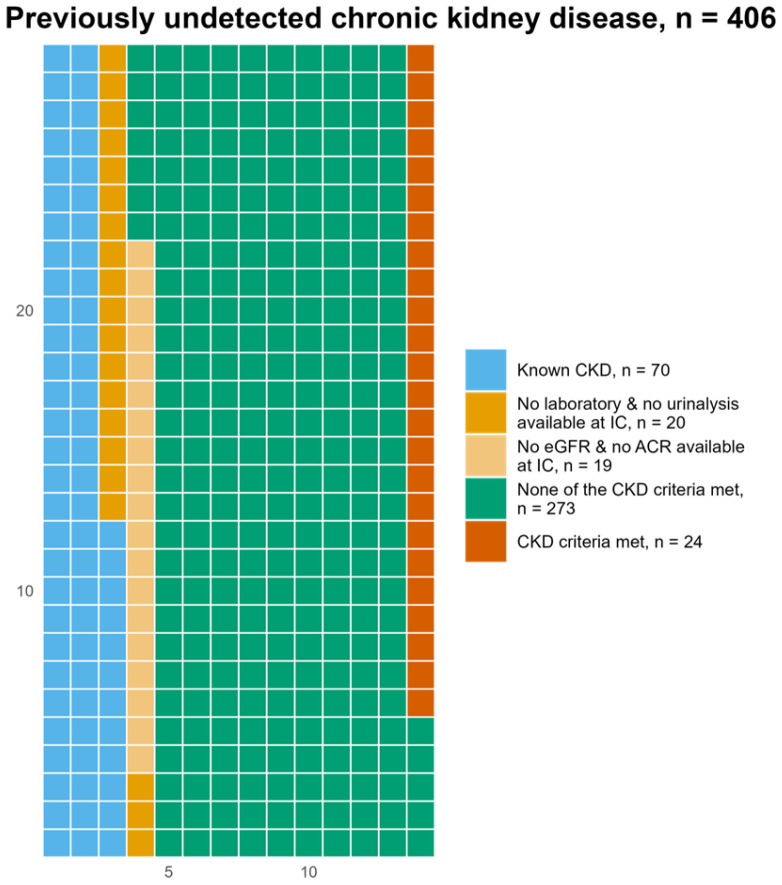
Waffle chart illustrating all previously undetected cases of chronic kidney disease (CKD) based on the above-mentioned criteria IC, initial consultation. Detailed definitions are provided in Section 2.

Among our participants, we identified 24 participants (5.9%) with previously undetected CKD. In total, 70 individuals (17.2%) had a previously known diagnosis of CKD. In 273 participants (67.2%) with available eGFR or ACR, none of the CKD criteria were met (Figure 5).

#### 3.3.3. Metabolic

**Figure 6:**
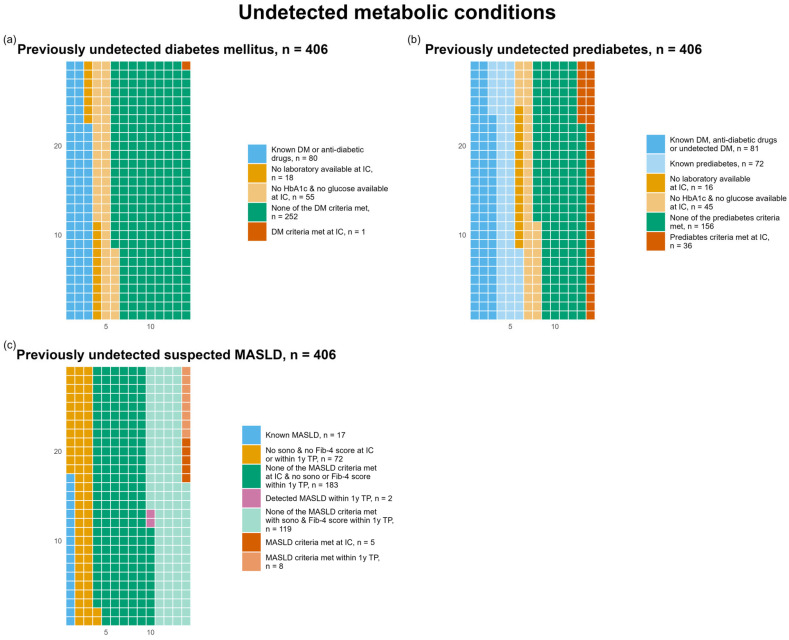
Waffle chart illustrating previously undetected diabetes mellitus (DM) based on the above-mentioned criteria (**a**); undetected prediabetes (**b**); and undetected suspected metabolic dysfunction-associated steatotic liver disease (MASLD), distinguishing detection at initial consultation (IC) and one-year data collection time point (1y TP) (**c**). Detailed definitions are provided in Section 2.

We found one additional case (0.2%) of previously undetected diabetes (Figure 6a) and 36 cases (8.9%) of previously undetected prediabetes (Figure 6b). A total of 80 participants (19.7%) had a known diagnosis of diabetes mellitus or were taking anti-diabetic drugs and 72 participants (17.7%) had a known diagnosis of prediabetes.

We detected 13 participants (3.2%) with previously undetected suspected MASLD, 5 cases (1.2%) at IC and 8 cases (2.0%) within the one-year TP. Meanwhile, during the one-year TP, two participants (0.5%) were diagnosed with MASLD during the regular clinical visits (Figure 6c). A detailed overview of the metabolic parameters and conditions can be found in Table A1 and Table A2.

## 4. Discussion

### 4.1. Fragmented Care in the Pre-CKM Era

This analysis is, to our knowledge, the first to investigate the diagnostic evaluation of patients attending a tertiary outpatient hypertension clinic in a European country in the context of the recently defined CKM syndrome by the AHA. Our findings demonstrate that, even before the introduction of the CKM framework, a more holistic evaluation of patients with CKM-related disorders would have been warranted, as CKM is increasingly recognized as a systemic and interrelated disorders process involving multiple organ systems. Several diagnostic blind spots became apparent. Although the CKM framework itself was introduced only recently, elements of cross-organ assessment, such as evaluation of cardiovascular risk factors and hypertension-related target organ damage, were already recommended in earlier clinical guidelines; our analysis therefore provides a quantitative assessment of how consistently such evaluations were implemented in routine practice.

One important observation is the relatively high proportion of participants with elevated lipoprotein(a) concentrations, where this measurement was available. Although selection bias cannot be excluded, it remains noteworthy that years after guideline recommendations, lipoprotein(a) was still not assessed in all patients with hypertension. Similarly, applying contemporary criteria revealed a considerable number of participants who fulfilled diagnostic criteria for previously undetected suspected heart failure, but only one patient had clinically apparent symptoms. This observation raises the question of whether earlier or more systematic biomarker-based screening strategies, such as routine NT-proBNP testing, which is inexpensive and widely available, could help identify patients with subclinical cardiac dysfunction in broader clinical settings. Echocardiographic data were also inconsistently available, further limiting structured cardiac evaluation.

Hepatic assessment emerged as another significant blind spot. Only a small fraction of participants underwent liver fibrosis evaluation despite the growing evidence linking MASLD with cardio–kidney–metabolic risk. In this context, non-invasive indices such as the FIB-4 score can serve as simple screening tools to identify individuals at increased risk of advanced fibrosis, although elevated values require further diagnostic evaluation and cannot establish a definitive diagnosis of MASLD.

The overall diagnostic landscape in this pre-CKM era setting was characterized by considerable heterogeneity. Not even essential parameters for evaluating cardiovascular, renal, and metabolic components, such as eGFR or ACR for hypertensive kidney damage or LDL-c for hypercholesterolemia [28], were assessed or available in all our participants. In some cases, even basic laboratory work-up was missing. Such inconsistency reflects the prevalent “organ-silo approach,” influenced both by pre-CKM guideline structures and by the absence of standardized multi-organ screening protocols. As a result, coexisting CKM-related disorders frequently remained undetected.

Among the 69 participants (17.0%) with available NT-proBNP at IC, 10 (14.5%) had values between >75 and ≤125 nmol/L, indicating possible cardiac stress, while 35 (50.7%) had values > 125 nmol/L, suggesting previously undetected suspected heart failure. As NT-proBNP was mainly measured when heart failure was clinically suspected, this finding likely underestimates the true prevalence of cardiac dysfunction in this cohort. At the same time, the retrospective application of sensitive biomarker thresholds may overestimate the prevalence of clinically manifest heart failure. Importantly, studies in cardiometabolic risk populations have shown that elevations in natriuretic peptides are frequently associated with structural cardiac abnormalities on imaging, supporting their role as markers of subclinical cardiac dysfunction [29]. The relevance of this observation therefore lies less in the precise prevalence estimate than in demonstrating that abnormal cardiac biomarkers were present in a substantial proportion of patients but were not consistently integrated into a structured diagnostic framework. Comparison of IC and one-year TP examinations suggested some improvement in diagnostic measures over time, yet a substantial proportion of participants still lacked CKM-related assessments. This inhomogeneous evaluation reflects the absence of standardized clinical pathways to guide which organ systems should be assessed in patients with CKM-related disorders. Consequently, opportunities for early detection and timely management were likely missed.

According to current guidelines, a significantly higher proportion of participants with a FIB-4 score ≥ 1.3 should have undergone abdominal ultrasound compared with what was observed in our cohort [30].

Taken together, our findings illustrate the fragmentation of care that preceded the re-emergence of CKM syndrome as an interconnected disorders framework and highlight the need for a more comprehensive, multidisciplinary evaluation of patients with cardiovascular, kidney, metabolic, and liver involvement.

### 4.2. Emerging Diagnostic Awareness Before the CKM Framework

Our data also reveal evolving diagnostic awareness over the examined years. Several CKM-relevant investigations increased between 2017 and 2022, although they were still far from universally implemented. For example, lipoprotein(a) measurement rose markedly from 0% in 2017 and 0.8% in 2019 to 23.7% in 2022. This trend may, at least in part, reflect the publication of the 2019 lipid guidelines recommending once-in-a-lifetime screening for lipoprotein(a) [21]. A similar pattern was observed for NT-proBNP testing, which increased from 10.1% in 2017 to 15.4% in 2019 and 23.7% in 2022. This temporal change coincides with growing evidence supporting the use of SGLT2 inhibitors in heart failure, broader recommendations for BNP-based screening in individuals with diabetes or stage A heart failure [31] and the emerging clinical recognition of the “heart stress” concept [24].

More modest improvements were observed in other diagnostic domains. The proportion of participants undergoing transthoracic echocardiography (TTE) increased only slightly over time (53.8% in 2017, 53.4% in 2019, and 57.7% in 2022), underscoring persistent heterogeneity in cardiovascular assessment. While these developments indicate incremental progress, they also highlight that diagnostic practice remained fragmentary and not guided by a systematic multi-organ screening framework.

Taken together, these temporal signals suggest that clinical awareness of CKM-related conditions was gradually increasing even before the AHA formalized the CKM concept. However, this emerging awareness did not yet translate into consistent implementation of comprehensive diagnostic pathways. Instead, the data illustrate a transitional period in which clinicians were slowly incorporating new recommendations and therapeutic considerations, while still operating largely within traditional organ-specific structures.

### 4.3. Actionable Lessons for Integrated CKM Screening

The high prevalence of previously undetected CKM-related disorders in our cohort was revealed through the diagnostic work-up available at the time, which itself was shaped by a traditional organ-specific structure of medical care and, historically, by limited therapeutic options beyond risk factor control. Similar observations have been made in other tertiary-care settings. Rumora et al. recently demonstrated that even within specialized cardiology environments, a substantial proportion of patients have unrecognized heart failure unless systematic biomarker- or imaging-based screening strategies are applied, underscoring that diagnostic blind spots are pervasive and not limited to primary care contexts [19].

The clinical landscape has since changed fundamentally. New therapeutic classes with proven multi-organ benefits, such as SGLT2 inhibitors, GLP-1 receptor agonists and the non-steroidal mineralocorticoid receptor antagonist finerenone, now offer effective interventions across cardiovascular, renal, and metabolic domains. Their availability has shifted clinical practice from the reactive treatment of established disorders to the proactive identification of subclinical organ dysfunction, as early detection directly influences therapeutic eligibility and long-term outcomes. Consequently, screening for CKM-related conditions is no longer merely a preventive exercise but a practical necessity for implementing organ-protective therapy.

These findings highlight the urgency of structured, multi-organ screening algorithms that translate therapeutic potential into real-world benefit. In our cohort, for example, 87 participants (21.4%) fulfilled criteria for previously undetected suspected heart failure, illustrating how a more systematic approach to early detection could meaningfully alter patient management. Nevertheless, the implementation of comprehensive screening strategies is often constrained by financial limitations, personnel resources, and local infrastructure. Any future screening model must therefore balance clinical relevance with practical feasibility to ensure that integrated CKM assessment can be embedded into routine care.

### 4.4. From Reflection to Implementation, Advancing Integrated CKLM Care

There is substantial potential to strengthen CKM-related diagnostics through the integration of simple and widely available laboratory tools, such as the FIB-4 score for liver fibrosis [32]. Furthermore, as discussed by other authors, expanding the CKM construct toward a cardio–kidney–liver–metabolic (CKLM) framework may more accurately capture the interconnected nature of organ involvement in these patients [9]. Achieving such an approach requires not only multidisciplinary education and training to promote a holistic clinical perspective, but also an interdisciplinary data structure and diagnostic framework capable of systematically documenting the cardio–kidney–liver–metabolic axis.

In reflection of these findings and in alignment with contemporary guidelines, structured diagnostic and reporting approaches may help promote systematic multi-organ assessment in patients with CKM-related conditions. Possible measures include the automated calculation of CKM-relevant indices, such as the Fib-4 score in routine laboratory panels to enhance clinician awareness; standardized electronic health record fields to ensure consistent documentation across organ domains; harmonized diagnostic and therapeutic standards defining screening intervals, thresholds, referral pathways and multidisciplinary review processes; and therapy-linked screening strategies to facilitate timely identification of organ damage relevant for the use of novel protective therapeutic agents. Such approaches may support the transition from fragmented organ-specific care toward a more integrated, evidence-based CKLM framework that embeds screening and prevention into routine clinical workflows. At our center, similar measures have recently been introduced as part of ongoing efforts to improve integrated cardio–kidney–liver–metabolic care.

Our data provide a snapshot of clinical reality before the CKM framework, illustrating both missed diagnostic opportunities and the path toward integrated CKLM care. They serve as a call to action, highlighting the need for structured multi-organ screening, particularly in patients with known CKLM-related disorders, given the substantial number of previously undetected conditions identified in this analysis. A more integrated diagnostic strategy may help close the current gap in early detection and enable targeted interventions to prevent disease progression and improve long-term outcomes.

These considerations may also inform the development of prospective initiatives aimed at monitoring real-world CKM-related care. One example is the prospective Swiss CKLM Registry, designed to monitor real-world clinical practice and support the transition from conceptual frameworks to practical implementation. As emphasized by current professional societies, well-designed clinical registries represent a crucial bridge between evidence and daily care. They enable continuous evaluation of outcomes, support the implementation of guideline-directed diagnostic and therapeutic strategies, and reveal gaps that would otherwise remain unnoticed. In the words of Peter Drucker, “if you can’t measure it, you can’t improve it.” By transforming the lessons of the pre-CKLM era into structured, data-driven workflows, such initiatives may contribute to refining clinical processes and advancing integrated CKLM care in clinical practice.

### 4.5. Limitations

This analysis has several limitations. First, as it was conducted as retrospective analysis in hypertensive patients attending a tertiary hypertension center, the generalizability of our findings may be limited, and extrapolation to broader or primary care populations should be made with caution. Patients referred to a tertiary center may represent a more complex or treatment-resistant population than those typically managed in primary care. At the same time, these individuals were not untreated patients but had already received medical care in various healthcare settings before referral, meaning that several opportunities for the detection of CKLM-related conditions may already have existed prior to their first consultation at our center. Second, some CKLM-related conditions may have been known to treating physicians but were not recorded or accessible in the local electronic health record. During the transition from paper-based to digital documentation, relevant clinical information may have been obtained but not systematically captured, particularly as standards for documenting CKLM-related measures were not yet established. This is illustrated by inconsistencies such as eGFR being available while ACR was missing in a subset of patients. This limitation reflects a common challenge of retrospective analyses based on routine clinical documentation, where information from external providers may not always be fully transferred or recorded in the local electronic health record. Furthermore, the reported prevalence of suspected heart failure may represent an overestimation. In our study, the classification was based exclusively on technical and biomarker-derived parameters, including TTE measurements and NT-proBNP levels. These parameters alone are not necessarily equivalent to a clinical diagnosis of heart failure. Importantly, we did not systematically assess clinical symptoms or signs typically required for diagnosis, such as dyspnea, reduced exercise tolerance, or peripheral edema. Therefore, our findings should be interpreted as indicating suspected heart failure rather than confirmed clinical disease of heart failure.

Third, diagnostic examinations performed outside our institution, such as by general practitioners or other hospitals, were not incorporated into our dataset and may have led to underestimation of known comorbidities. In addition, not all patients had a 6- or 12-month TP, and the predefined time windows for data extraction may have contributed to incomplete retrieval of available diagnostic information. Because diagnostic testing was not systematically performed at baseline in all participants, conditions detected during the one-year observation period may represent either previously unrecognized disease or pathology that became apparent during subsequent clinical evaluation. The strict criteria applied to identify previously undetected disorders may also have resulted in an overestimation of their prevalence.

Finally, as the CKLM concept had not yet been defined at the time these patients were evaluated, several parameters relevant to contemporary CKLM assessment, including lipoprotein(a) or Fib-4, were not routinely measured. While this represents a limitation of the dataset, it also reflects real-world clinical practice in the pre-CKLM era and highlights the heterogeneity of diagnostic approaches at that time, a finding that ultimately strengthens the relevance of our analysis.

## 5. Conclusions

Greater awareness of CKLM-related comorbidities in patients already affected by a CKLM-related condition is urgently needed. Routine and systematic screening for additional cardiovascular, kidney, metabolic, and hepatic involvement should be considered to avoid missing clinically relevant and potentially treatable disorders. Failure to detect such coexisting conditions represents a missed opportunity to initiate organ-protective therapies and possibly to improve long-term outcomes. Physicians should therefore not only perform appropriate diagnostic investigations but also interpret findings within a holistic, multi-organ framework to guide timely and targeted interventions.

Further studies are required to determine the clinical and economic impact of structured multiorgan screening strategies and to assess whether a more integrated approach to CKLM syndrome management can effectively improve patient outcomes. These efforts will be essential to develop practical, evidence-based guidelines that support clinicians in delivering comprehensive CKLM care.

## Figures and Tables

**Table 1:** Initial consultation characteristics.

Characteristic	Overall (*n* = 406)
Sex (male); *n* (%)	229 (56.4)
Age, years; mean (SD)	57 (15.6)
Height, cm; mean (SD)	171.0 (10.5)
Weight, kg; mean (SD)	83.7 (19.9)
BMI, kg/m^2^; mean (SD)	28.5 (5.7)
AOBP systolic, mmHg; mean (SD)	136 (21.0)
AOBP diastolic, mmHg; mean (SD)	82 (11.6)
Number of daily pills; mean (SD)	5.2 (4.7)
Number of total medication burden; mean (SD)	4.5 (3.4)
Number of antihypertensive pills; mean (SD)	2.5 (2.0)
Number of antihypertensive medication burden; mean (SD)	3.1 (2.2)
Nonsmoker; *n* (%)	204 (50.9) (*n* = 401)
Previous smoker; *n* (%)	104 (25.9) (*n* = 401)
Active smoker; *n* (%)	93 (23.2) (*n* = 401)
CAD; *n* (%)	66 (16.3)
CVI/TIA; *n* (%)	54 (13.3)
PAD; *n* (%)	24 (5.9)
Aortic aneurysm; *n* (%)	11 (2.7)
Atrial fibrillation; *n* (%)	29 (7.1)
Chronic inflammatory disease; *n* (%)	47 (11.6)
Mental illness history; *n* (%)	87 (21.4)
Documented positive family history of CVD; *n* (%)	87 (21.4)
Documented family history of DM; *n* (%)	61 (15.0)
No TP at 6 and 12 months	201 (49.5)
TP at 6 or/and 12 months	205 (50.5)
TP at 6 months; *n* (%)	149 (36.7)
TP at 12 months; *n* (%)	145 (35.7)

Data are mean (±standard deviation) or counts (percentage), as appropriate. Detailed definitions are provided in Section 2. Medical treatment was characterized by medication burden, with total medication burden defined as the number of prescribed medication classes, antihypertensive medication burden as the number of antihypertensive drug classes, and the number of antihypertensive pills as the actual daily pill count of antihypertensive drugs. AOBP, automated office blood pressure; BMI, body mass index; CAD, coronary artery disease; CVD, cardiovascular disease; CVI, cardiovascular insult; DM, diabetes mellitus; PAD, peripheral artery disease; TIA, transient ischemic attack; TP, data collection time points.

## Data Availability

The data supporting the findings of this study are available from the corresponding author upon reasonable request. Due to ethical and data protection regulations, the data are not publicly available.

## Abbreviations

The following abbreviations are used in this manuscript:

ACR	Albumin-to-creatinine ratio
AHA	American Heart Association
ALT	Alanine aminotransferase
AOBP	Automated office blood pressure
AST	Aspartate aminotransferase
BMI	Body Mass Index
CKD	Chronic Kidney Disease
CKM	Cardiovascular–kidney–metabolic
CKLM	Cardio–kidney–liver–metabolic
CKLMD	Cardio–kidney–liver–metabolic disease
CVD	Cardiovascular Disease
DPP-4	Dipeptidyl peptidase 4
eGFR	Estimated glomerular filtration rate
Fib-4	Fibrosis-4
GLP-1	Glucagon-like peptide-1
HbA1c	Hemoglobin A1c
IC	Initial consultation
KDIGO	Kidney Disease: Improving Global Outcomes
LAVI	Left atrial volume index
LDL-c	Low-density lipoprotein cholesterol
LLD	Lipid-lowering drugs
LVEF	Left ventricular ejection fraction
LVMI	Left ventricular mass index
MASLD	Metabolic Dysfunction-associated Steatotic Liver Disease
NT-proBNP	N-terminal pro–B-type natriuretic peptide
PCSK9	Proprotein convertase subtilisin/kexin type 9
SD	Standard deviation
SGLT2	Sodium-glucose transport 2
SMuRFs	Standard Modifiable Risk Factors
TP	Data collection time point
TR	Tricuspid regurgitation
TTE	Transthoracic echocardiography

## Appendix A

**Table A1:** Overview of assessment and pathological findings of diagnostic parameters at initial consultation (IC) and within one-year data collection time point (1y TP).

Diagnostic Parameter	Patients Assessed, *n* (%)	Patients Not Assessed, *n* (%)	Pathological Findings, *n* (%)
NT-proBNP, IC	69 (17.0)	337 (83.0)	45 (11.1)
NT-proBNP, within 1y TP	117 (28.8)	289 (71.2)	91 (22.4)
TTE, IC	224 (55.2)	182 (44.8)	62 (15.3)
TTE, within 1y TP	287 (70.7)	119 (29.3)	79 (19.5)
Lipoprotein(a), IC	38 (9.4)	368 (90.6)	14 (3.5)
Lipoprotein(a), within 1y TP	55 (13.5)	351 (86.5)	21 (5.2)
LDL-c, IC	291 (71.7)	115 (28.3)	123 (30.3)
LDL-c, within 1y TP	320 (78.8)	86 (21.2)	135 (33.3)
eGFR, IC	349 (86.0)	57 (14.0)	67 (16.5)
eGFR, within 1y TP	369 (90.9)	37 (9.1)	83 (20.4)
ACR, IC	289 (71.2)	117 (28.8)	78 (19.2)
ACR, within 1y TP	324 (79.8)	82 (20.2)	96 (23.6)
Glucose, IC	180 (44.3)	226 (55.7)	6 (1.5)
Glucose, within 1y TP	215 (53.0)	191 (47.0)	13 (3.2)
HbA1c, IC	291 (71.7)	115 (28.3)	44 (10.8)
HbA1c, within 1y TP	315 (77.6)	91 (22.4)	55 (13.5)
Fib-4 score, IC	295 (72.7)	111 (27.3)	4 (13.5)
Fib-4 score, within 1y TP	330 (81.3)	76 (18.7)	8 (2.0)

Data are counts (percentage). Percentages were calculated based on the total study population. Diagnostic assessments were considered “performed” when the respective test was documented during the index evaluation at the hypertension center. “Not assessed” indicates that the specific parameter was not measured during the index evaluation at our center. In some cases, testing may not have been repeated because the respective condition had been previously diagnosed and documented in the medical record. Pathological findings were defined according to the diagnostic thresholds specified in Section 2. Therefore, “not assessed” does not necessarily indicate absence of prior evaluation but reflects testing not performed or not repeated during the index assessment. ACR, albumin-to-creatinine ratio; eGFR, estimated glomerular filtration rate; Fib-4, fibrosis-4; HbA1c, hemoglobin A1c; LDL-C, low-density lipoprotein cholesterol; NT-proBNP, N-terminal pro–B-type natriuretic peptide; TTE, transthoracic echocardiography.

**Table A2:** Previously undetected conditions at initial consultation (IC) and for previously undetected suspected heart failure and previously undetected suspected MASLD/liver fibrosis within one-year data collection time point (1y TP).

Condition	Patients Assessed, *n* (%)	Patients Not Assessed, *n* (%)	Known Condition, *n* (%)	Previously Undetected Condition, *n* (%)
Suspected heart failure, IC	232 (57.1)	156 (38.4)	18 (4.4)	62 (15.3)
Suspected heart failure, within 1y TP	291 (71.7)	96 (23.6)	19 (4.7)	87 (21.4)
Hypercholesterolemia, IC	98 (24.1)	47 (11.6)	261 (64.3)	17 (4.2)
CKD, IC	297 (73.2)	39 (9.6)	70 (17.2)	24 (5.9)
Suspected MASLD/liver fibrosis, IC	281 (69.2)	108 (26.6)	17 (4.2)	5 (1.2)
Suspected MASLD/liver fibrosis, within 1y TP	315 (77.6)	72 (17.7)	19 (4.7)	13 (3.2)
Diabetes mellitus, IC	253 (62.3)	73 (18.0)	80 (19.7)	1 (0.2)

Data are counts (percentages). Percentages were calculated based on the total study population. Previously undetected conditions were defined as diagnoses established during the index evaluation without prior documentation in the patient’s medical record. Definitions of CKM-related conditions are provided in Section 2.5. CKD, chronic kidney disease; MASLD, metabolic dysfunction-associated liver disease.
